# Deciphering the role of natural variation in age-related protein homeostasis

**DOI:** 10.1186/1741-7007-11-102

**Published:** 2013-09-30

**Authors:** Matt Kaeberlein

**Affiliations:** 1 Department of Pathology, University of Washington, 1959 NE Pacific Street, D-514, Seattle, WA 98195-7470, USA

## Abstract

Understanding the genetic basis of age-related diseases is a critical step toward developing therapies that promote healthy aging. Numerous genes have been identified that modulate lifespan, but the influence of natural variation in aging has not been well studied. A new report utilizing a transgenic protein aggregation model in *Caenorhabditis elegans* has provided important tools and insights into the relationship between natural genetic variation, protein aggregation, and age-related pathology.

See research article: http://www.biomedcentral.com/1741-7007/11/100

## Commentary

Age is the single greatest risk factor for a majority of the major causes of death and disability in developed countries [1]. As populations in these nations continue to achieve greater life expectancies, the increase in prevalence of age-associated diseases presents a particularly challenging problem. One important class of age-associated diseases, including Alzheimer’s and other neurodegenerative diseases, can be attributed to a failure to maintain protein homeostasis resulting in the accumulation of toxic proteins over time. Identifying the genetic factors that modulate why this happens in the context of an aging organism and, equally importantly, why it does not happen the same way in every individual, is essential for developing therapies that can delay or prevent these disorders. In this issue of *BMC Biology*, Gidalevitz *et al*. [2] characterize a series of genetically dissimilar transgenic *C. elegans* strains designed to assess the contribution of natural genetic variation to age-associated protein aggregation and toxicity. In the process they discovered that not only does natural genetic variation dramatically influence the propensity of proteins to aggregate, but that aggregation and toxicity are not strongly correlated.

*C. elegans* has been utilized extensively as a model system to study human diseases of proteotoxicity, a term used to refer to the detrimental effects of misfolded or aggregated proteins on cells. Human proteotoxic diseases include prion disorders, motor neuron disease, and various amyloidoses, as well as Alzheimer’s, Huntington’s and Parkinson’s diseases. In addition to the powerful genetic tools available to *C. elegans* researchers, several features make *C. elegans* a particularly useful model in this regard. These include the relative ease of making transgenic lines, the fact that adult *C. elegans* are primarily post-mitotic (with the exception of the germ line), and that an adult lifespan is two to three weeks for animals maintained in the lab. These features make it possible to express an aggregation-prone or toxic peptide of choice transgenically, either broadly or in a cell-type restricted manner, and, if fused to a fluorescent reporter such as GFP (green fluorescent protein) or YFP (yellow fluorescent protein), watch the formation of protein aggregates as the animals get older. Precisely this approach has been taken with several human disease-related peptides, including beta amyloid, tau, TDP-43, and alpha synuclein [3]. Although each toxic peptide has its own unique features and kinetics of toxicity, there are striking similarities between these different transgenic lines, including common genetic modifiers and clear age-associated pathology. In a majority of cases, toxic peptides have been expressed either neuronally or in body wall muscle cells, with body wall muscle expression yielding a robust age-associated paralysis phenotype, while neuronal expression tends to yield more subtle age-associated changes in movement or behavior, with accompanying cell loss in some cases.

Among the most utilized of these strains are a series of transgenic lines expressing polyglutamine peptides of varying length fused to YFP that were generated by the Morimoto lab [4,5]. Polyglutamine expansions within different proteins underlie several human diseases, including Huntington’s, spinobulbar muscular atrophy, dentatorubral-pallidoluysian atrophy and several spinocerebellar ataxias. As in the human diseases, the polyglutamine-YFP peptides show age-dependent aggregation, with the rapidity of aggregation and cytotoxicity correlating strongly with the length of the polyglutamine repeat. Although it remains unclear how well these *C. elegans* strains recapitulate other major aspects of human polyglutamine diseases, there is no question that they have been, and continue to be, useful tools for understanding the genetic and molecular mechanisms of proteotoxicity.

One of the primary discoveries to come from studies of transgenic polyglutamine toxicity, as well as the other proteotoxicity models, in *C. elegans* is that the same genetic and environmental features that modulate aging also modulate resistance to proteotoxic stress. This makes intuitive sense, since proteotoxic diseases are generally age-associated; however, it also strongly supports the hypothesis that loss of protein homeostasis may be a contributing causal factor for aging, particularly in post-mitotic cells and tissues. Consistent with this idea, studies have shown that three of the best-studied longevity pathways in *C. elegans*, the insulin-like signaling pathway [6], dietary restriction [7], and the hypoxic response pathway [8], all similarly modulate resistance to polyglutamine toxicity. Several additional important observations have also come from these and similar studies.

The first of these important observations is that aggregation does not necessarily reflect toxicity, at least for muscle cells. At least two lines of evidence support this idea. First, when polyglutamine is expressed in body wall muscle cells, dietary restriction extends lifespan and dramatically suppresses toxicity (defined here by onset of age-associated paralysis), without reducing the number or distribution of large polyglutamine aggregates [7]. Second, reduced signaling through the insulin signaling pathway appears to suppress proteotoxicity by two distinct mechanisms: heat shock factor one (HSF-1) promotes disaggregation of toxic intermediates while the FOXO transcription factor DAF-16 promotes the formation of large molecular weight aggregates, which are apparently no longer toxic [9].

A second important observation is that exogenous proteotoxicity can affect healthspan without affecting lifespan. Expression of a 40-glutamine-repeat peptide fused to YFP in body wall muscle cells causes nearly all animals to become paralyzed by around 2 weeks of adulthood without significantly affecting adult lifespan [7].

And a third important observation is that protein homeostasis declines during normal aging. Even in animals that do not express a toxic transgenic peptide, there is now substantial evidence that misfolded and aggregated proteins accumulate with age [10]. This has led to the hypothesis that a disease-causing aggregation-prone mutant protein can serve as a ‘seed’ to initiate a toxic cascade of protein misfolding and aggregation that amplifies the underlying age-associated increase in proteotoxic stress.

Despite these insights into the role of protein homeostasis in disease, one missing link has been connecting natural variation to protein homeostasis and aging. The vast majority of genetic studies in this area have been performed using RNA interference knockdown or mutations (often gene deletions) derived from chemical mutagenesis. Gidalevitz *et al*. [2] have taken the first step in addressing this limitation by crossing the aforementioned muscle-specific 40 polyglutamine-YFP peptide into three genetically dissimilar wild *C. elegans* strains and then creating a series of recombinant inbred lines. Just in the process of creating these lines, the authors noted that genetic background strongly influences the phenotypic response (degree and rate of paralysis) to expression of the toxic peptide, as well as which subsets of muscle cells are most likely to form aggregates. In addition, they provided further confirmation that the formation of large aggregates does not always correlate strongly with toxicity, and that this relationship is likely influenced by the underlying genetic background.

Going forward, these new transgenic tools will help define the role of natural genetic variation in protein homeostasis and aging. It will be of particular interest to see which new genes are implicated in this process and whether they fall into the established pathways or define new and previously unsuspected mechanisms. It will also be of interest to determine whether the same genes modulate age-related diseases in mammals. Ultimately, as we learn more about how and why protein homeostasis breaks down during aging it should be possible to slow, or perhaps even prevent, that breakdown.
